# A Sensor Control Model for Cabled Seafloor Observatories in the East China Sea

**DOI:** 10.3390/s18093027

**Published:** 2018-09-10

**Authors:** Yang Yu, Huiping Xu, Changwei Xu

**Affiliations:** 1 State Key Laboratory of Marine Geology, Tongji University, Shanghai 200092, China; xcw@tongji.edu.cn; 2 Institute of Deep-Sea Science and Engineering, Chinese Academy of Sciences, Sanya 572000, China; xuhp@sidsse.ac.cn

**Keywords:** seafloor observatories, control system, sensor control, layered model

## Abstract

Seafloor observatories enable real-time, continuous and long-term observations that promise major breakthroughs in ocean sciences. The effort to dynamically control in situ sensor systems performing individual and cooperative observation tasks is both a challenge and a guarantee for the stable operations of functional observatories. However, current sensor control systems for seafloor observatories are application-oriented and focus on specific engineering requirements rather than general model research. In this paper, a sensor control model was proposed to provide a theoretical specification for designing, developing and deploying a sensor control system for cabled seafloor observatories. The model abstracted the sensor control as a standardized bidirectional information flow process and accordingly structured the control system into the application layer, the service layer, the networking layer, and the sensing layer. The layered functions and interactions between adjacent layers in return realized this information process. A prototype control system was designed and developed, the monitoring architecture of which was derived from one of the deployment scenarios of the sensor control model. The prototype system was tested for its plug-and-play enablement through a series of trials such as tank tests and shallow sea trials. It was put into service for the operational Xiaoqushan Seafloor Observatory and was consistently functioning and presenting satisfactory practical performance in accordance with all the requirements laid for the project.

## 1. Introduction

Long-term and in situ observations have dominated oceanographic studies in recent decades [1]. Emerging in response to this trend, seafloor observatories are widely recognized as the third observation platform for geosciences following remote sensing [2]. Seafloor observatories are designed to put all the sensor equipment under the sea, which can realize continuous power supply and real-time bidirectional data transmission between in situ sensor systems and the control center on shore [3]. In this way continuous and multidisciplinary measurements are made within the whole water body, which contribute to a better understanding of the fine structure and various processes in the ocean interior. Seafloor observatories mark new research approaches for several branches of ocean sciences [4].

Governments and scientists have made great efforts in researching seafloor observatory systems. Many projects and programs on seafloor observatories have been implemented. The USA was the earliest and most advanced county in developing seafloor observatories, owning several like MARS and OOI [5,6,7,8]. OOI comprises three scales of observatory systems (coastal, regional and global) that are integrated by an overarching cyberinfrastructure, transitioning the oceanographic community from expedition-based data gathering to persistent and controllable observations. In Europe, undersea long-term observations started early and constructions of seafloor observatory systems have centered on GEOSTAR and SN-1 in recent years [9,10]. Japan focuses on earthquake monitoring in terms of establishing seafloor observatories, with GeO-TOC, DONET and observatories to be built all deployed for earthquake early warning [11,12]. Canada has led international seafloor observatory research with the successively built VENUS and NEPTUNE, which are now integrated into Ocean Networks Canada (ONC) [13]. ONC enables evidence-based decision-making on ocean management and environmental protection using cabled observatories, remote control systems, interactive sensors, and big data management. China is actively preparing its China National Scientific Seafloor Observatory (CNSSO), with the Xiaoqushan Seafloor Observatory in the East China Sea (ECS) as the first trial [14,15,16].

The effort to dynamically control in situ sensor systems performing individual and cooperative observation tasks is both a challenge and a guarantee for the stable operations of functional observatories. The sensor control system remotely controls the working state of various submarine sensor equipment, ensuring that the observed raw data meet with real-time observation demands of seafloor observatories. The guaranteed data source provides subsequent information service platforms with the information base for data processing, data storage, data analysis, data application, and close-loop observation. As stated above, current sensor control systems for seafloor observatories are application-oriented and focus on specific engineering requirements rather than general model research [17,18,19,20,21,22,23,24,25,26,27]. In this paper, we propose a sensor control model for cabled seafloor observatories to provide a theoretical specification for designing, developing and deploying a sensor control system.

The paper is organized as follows: in Section 2, the information-oriented sensor control model is introduced in terms of model theory, functional layers, layer interfaces and deployment scenarios. In Section 3, the prototype control system is described about its plug-and-play enablement and practical applications in the Xiaoqushan Seafloor Observatory. In Section 4, contributions of the sensor control model are discussed and concluded.

## 2. Model Design

### 2.1. Related Work

**Figure 1:**
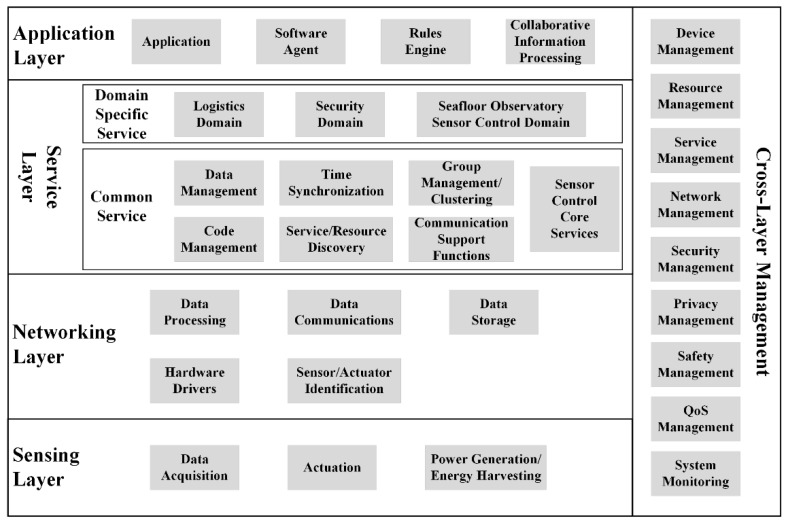
Functional Entities for Controlling Seafloor Observatory Sensor Networks.

One of the most important related advances [28,29] is the ISO/IEC 29182 Sensor Network Reference Architecture (SNRA). The purpose of this international standard is to facilitate the design and development of sensor networks, improve interoperability of sensor networks and make sensor network components plug and play. This SNRA contains a set of domains concerned with gathering raw data from physical environment, processing raw data into information, and delivering information to users which can be a human or a machine/software (e.g., automated command and control system). The sensing domain interfaces with sensors, gateways, and other entities such as storage devices in a sensor network. It receives data from the sensor network and transmits to the service domain via the network domain per requests made by users. Additionally, the sensing domain can have capabilities to process measurements such as raw data from sensors in the sensor network via local area network and/or wide area network. The network domain provides data/information links between the sensing domain and the service domain. The service domain hosts various applications that provide requested services by users. To perform the required applications, the service domain can also support various data processing capabilities for data and/or information from the sensing domain via the network domain. All the functional entities within these three domains can also be grouped into two categories. The Data, Information and Communication Group is responsible for data processing, information generation and communications of the data/information generated between the domains and to data/information requesters. The Control and Management Group is responsible for managing entities within each domain. As a hybrid and extension of the ISO SNRA, functional entities for controlling a seafloor observatory sensor network are proposed as shown in Figure 1.

Another key advance [30] is the dependable embedded wireless infrastructure (DEWI) high-level architecture (HLA). The core concept of the DEWI solution is the DEWI Bubble, which is a logical entity and high-level abstraction of a wireless sensor network (WSN). The layer model of DEWI HLA contains three levels. Level 0 is the intra-WSN layer, of which each WSN consists of a set of sensors or actuators also known as DEWI Nodes controlled by the corresponding WSN Gateway (WGW). Level 1 is the intra-Bubble layer responsible for interconnecting several WSNs and WGWs to their corresponding DEWI Bubble Gateway. Level 2 is the layer of the DEWI HLA in charge of the communication between DEWI Bubbles and external users, enabling the interoperability and cross-domain application development. 

Besides the two related advances mentioned above, the Internet of Underwater Things (IoUT) architecture [31] could also be a useful reference for proposing the sensor control model for cabled seafloor observatories. IoUT is defined as a worldwide network of smart interconnected underwater objects with a digital entity, the architecture of which is divided into three layers. The perception layer identifies underwater objects and collects information from in situ marine environment. Components of the perception layer include underwater sensors, underwater vehicles, monitoring stations, data storage tags, acoustic/radio/PIT tags and hydrophones/receivers/tag readers. The network layer processes and transmits the information obtained from the perception layer. It is formed by a converged network of wired/wireless privately owned networks, Internet, network administration systems, cloud computing platforms, and so on. The application layer is a set of intelligent solutions that apply the IoUT technology to meet with the user needs. 

### 2.2. Functional Layers

The sensor control model for cabled seafloor observatories was proposed to rationalize and standardize a sensor control system, minimizing overall effects when some part of the system changed. The model abstracted the remote and dynamic sensor control as a standardized bidirectional information flow process, which was further decomposed into three independent information interaction sub-processes and was realized by interactions between functionally divided layers of the model.

**Figure 2:**
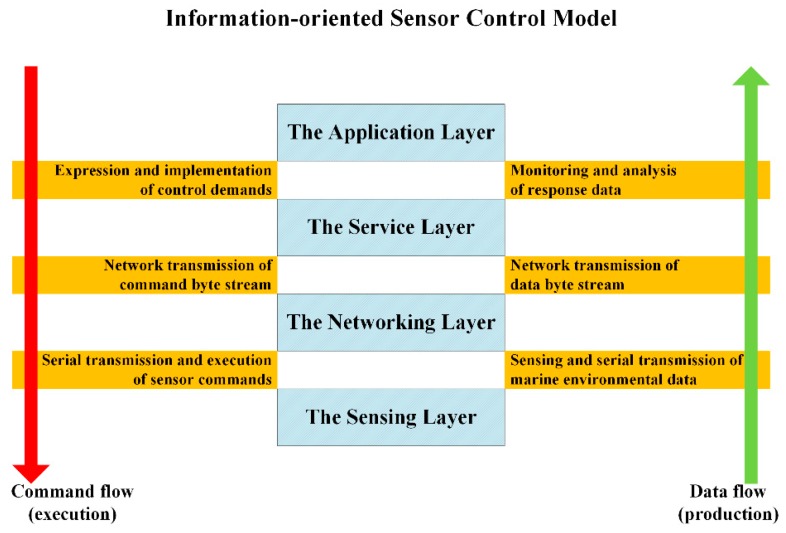
Information-oriented Sensor Control Model.

Based on the communication paths of both command flow and data flow within the sensor control system, the model structured the control system into the application layer, the service layer, the networking layer, and the sensing layer. Layers were relatively functionally independent with interfaces between them defined in a standard way. Each layer used services from its adjacent bottom layer and provided services to its adjacent upper layer. Layers interacted via standard interfaces to fulfill three main information exchange processes of the two information flows within the sensor control system (as shown in Figure 2).

#### 2.2.1. The Sensing Layer

The sensing layer executed observation plans (control commands) and sensed the dynamic marine environment, forming the information base for the control model. The sensing layer commonly referred to various sensor systems, such as multidisciplinary marine sensors, oceanographic instrumentation and equipment of various spatial and temporal scales, and ocean wireless sensor networks. According to the control commands passed by the networking layer, the sensing layer used corresponding sensors to obtain analog signals of in situ marine environment measurements and converted them into digital signals for the upper networking layer.

#### 2.2.2. The Networking Layer

The networking layer converted in situ sensors into sensing endpoints in the observatory network, and relayed bidirectional information between the service layer and the sensing layer. The networking layer established the one-to-one mapping between network information of IP/port number and physical serial port number, relaying control commands from the service layer to corresponding sensors in the sensing layer and transmitting all the collected observation data to the service layer. In this way, the networking layer enabled the conversion from in situ observatory sensors to networked sensing resources capable of remote communication and interactive access. A typical demonstration was the design of general junction box (GJB) in cabled seafloor observatories. GJB established the one-to-one mapping between serial communication and network communication in the following way: on the one hand, GJB used RS232/485 serial communication to collect all the observation data sampled by various marine sensors; on the other hand, GJB interfaced with the remote control client by means of IP/port addressing and relayed various control commands from the control system of upper layers to corresponding sensors for dynamic sensing of in situ marine environment. The networking layer logically isolated the sensing layer and the service layer. Once observatory sensors were changed in the sensing layer, upgrades were only needed for corresponding software and hardware modules in the networking layer while the way that the service layer interacted with the sensing layer via the networking layer was not affected. To be specific, the service layer could establish layered connections and send control commands to or receive observation data from in situ sensors of the sensing layer in the original way (e.g., IP/port number addressing).

#### 2.2.3. The Service Layer

The service layer provided a set of functions related to sensor control, and served as the functional center of the whole control model. The core of the service layer was a group of services to manage and process various types of information, encapsulated in such as dynamic link libraries and web services. The service mode was dependent on the architecture protocol between the application layer and the service layer and can be implemented as client software, web application systems, and so on. The service layer, on the one hand, backed up the real-time observation data and status monitoring data from the networking layer for further processing. On the other hand, the service layer translated sensor control demands of the application layer into various functional services to pass control commands to the networking layer. The service layer logically isolated the networking layer and the application layer. When the operating parameters of the networking layer were changed, only the corresponding functional services in the service layer needed upgrading while how the application layer interfaced with the networking layer was not affected. In other words, application requirements including sensor control and data management were expressed and implemented in the original way (e.g., calling component interfaces).

#### 2.2.4. The Application Layer

The application layer was an engineering-oriented layer that used different information to meet sensor control requirements laid for the project. Based on data monitoring and analysis results, the application layer passed sensor control demands to the service layer and obtained various sensor response information. The application layer was commonly expressed via functional interfaces of various application systems. One demonstration was the control system for cabled seafloor observatories in the East China Sea (ESOCS). By calling and integrating related services, ESOCS managed to remotely control in situ marine sensors in terms of connection status, working state, and other related sensor control functions. The interface between the application layer and the service layer was complicated, the architecture of which would be in accordance with specific engineering requirements.

### 2.3. Interfaces between Adjacent Layers

The sensor control model for cabled seafloor observatories not only divided sensor control systems into functional layers, but also defined interface parameters and functional services between the adjacent upper layer and the adjacent bottom layer.

**Figure 3:**
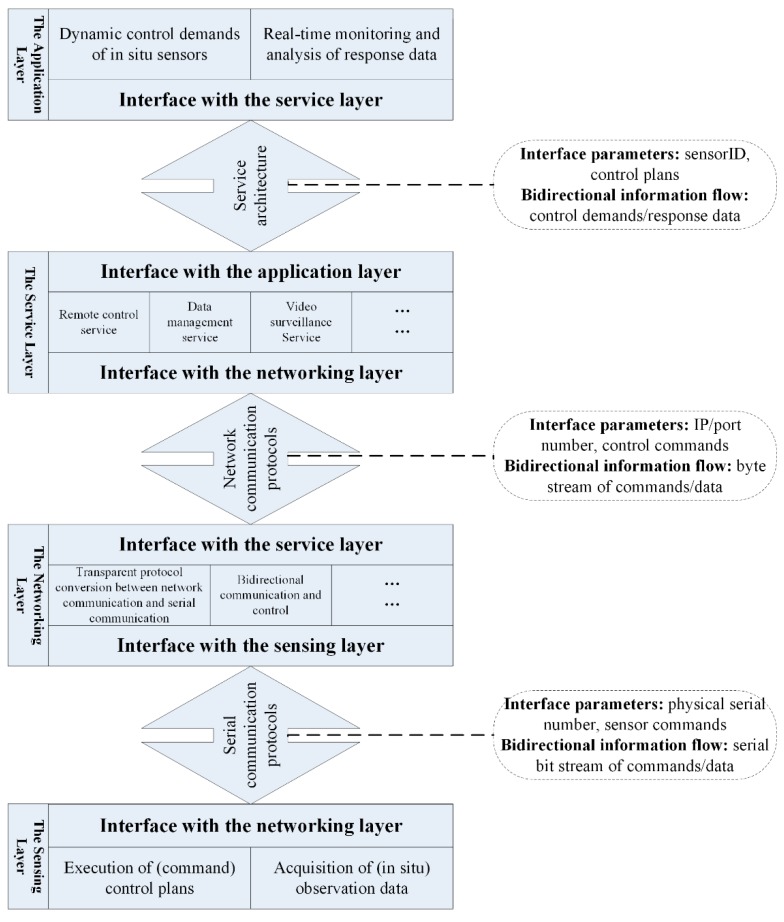
Interfaces between Adjacent Layers.

Serial communication between the sensing layer and the networking layer was selected, the interface standards of which included RS232 and RS485. Based on these serial communication interfaces, sensing equipment in the two layers carried out bidirectional data interactions in accordance with the protocols of the different sensor manufactures. As shown in Figure 3, interface parameters passed from the networking layer to the sensing layer included physical serial number and sensor control commands. Marine sensors in the sensing layer received and executed the serial control commands, returning responsive observation data and status data in ACSII format to the networking layer. Furthermore, the networking layer was in charge of data aggregation, protocol conversion and data transmission.

The interface between the networking layer and the service layer complied with TCP/IP communication protocol, the communicated data types of which included scientific observation data, sensor control commands and equipment status data. As shown in Figure 3, the service layer sent control commands to the networking layer via IP/port number addressing to manage or adjust the working state of various sensors. The networking layer in return collected all the serially transmitted data and converted them into network data stream for the service layer to store and analyze. In terms of the downlink, the networking layer converted control commands transmitted over the network into serially transmitted information and relayed them to the corresponding in situ marine sensors. As for the uplink, the service layer called related functional services to analyze and visualize different types of sensor information for the application layer, thus meeting the sensor control demands posed by the application layer.

The interface between the service layer and the application layer, which might be client-server architecture (C/S) or browser-server architecture (B/S), was complicated. Interface parameters passed from the application layer to the service layer were sensor ID and the control plan for cabled seafloor observatories. The service layer called related services to express the application requirements of the application layer as formatted control commands and to pass them to the networking layer. Meanwhile, the service layer returned various types of response information to the application layer. The interaction information between the service layer and the application layer included real-time observation/control demands of the control system, responsive observation data and equipment status data, and so on.

### 2.4. Deployment Scenarios

**Figure 4:**
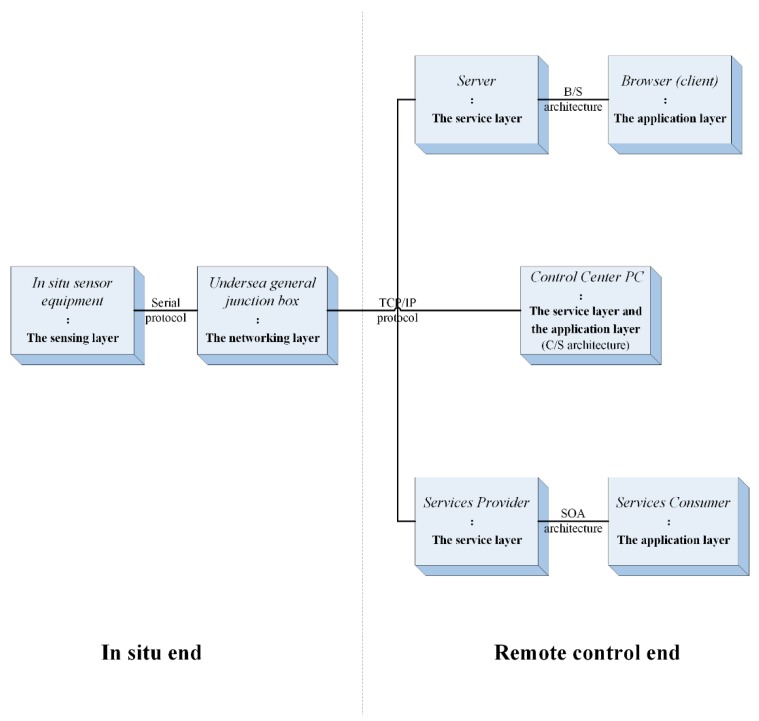
Deployment Scenarios of the Model.

The layers of the sensor control model could be deployed flexibly along the path where the bidirectional information flow was transmitted, thus forming different deployment scenarios in accordance with specific control demands or system requirements. In terms of ESOCS, three deployment scenarios derived from the control model were proposed in Figure 4. These deployment scenarios shared common features that the sensing layer referred to various marine sensors and the networking layer was implemented via the undersea general junction box. In the first scenario, the application layer and the service layer were implemented with the same monitoring PC at the control center. In the second scenario, the application layer was implemented in the client machine (browser) while the service layer was implemented in the server machine. In the third scenario, the application layer and the service layer were implemented in different physical environments and abided by the service-oriented architecture. A prototype experiment of ESOCS was conducted with the first deployment scenario, which was introduced in detail in the next section.

## 3. Prototype System Experiment

### 3.1. Model-Derived Monitoring Architecture

**Figure 5:**
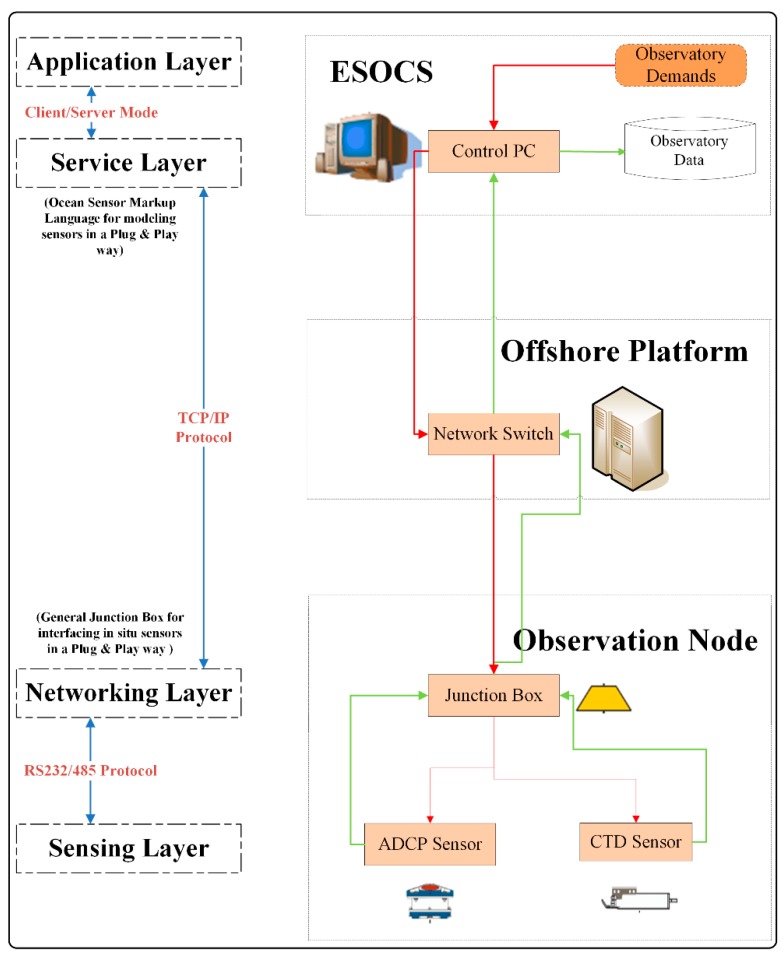
Monitoring Architecture for ESOCS.

A prototype sensor control system, namely ESOCS, was designed and developed. As stated in Figure 5, the monitoring architecture of ESOCS was derived from the first deployment scenario of the sensor control model. The architecture contained three components (Observation Node, Offshore Platform, and ESOCS) and enabled bidirectional information flow of observation data and control commands. The green flow contained in situ observation data gathered from cabled seafloor observatories in the ECS; while the red flow stood for types of commands sent to control undersea sensors within the Observation Node in accordance with real-time monitoring schedules. The architecture related to the layered control model in the following way. The application layer and the service layer were deployed within ESOCS while the networking layer and the sensing layer were respectively distributed within undersea general junction box and in situ sensor equipment of the Observation Node. 

The in situ junction box, one ADCP sensor, and one CTD sensor, constituted the Observation Node of cabled observatories. Observation data captured by deployed sensors was first transmitted to the communication control module of the junction box for encoding, and then forwarded to the network switch on the offshore platform for buffering via the submarine electro-optic cable under TCP/IP protocol. Upon arriving at the switch, all collected data entered the remote transmission phase and were received and stored by ESOCS running at the control center in a real-time way (as the green information flow in Figure 5). All the received raw data was stored in the database and simultaneously backed up in notepad (.txt) files in consideration of the system’s current demands. ESOCS was, on the one hand, responsible for receiving real-time data packets and preparing them for further data processing or application. On the other hand, this system was mainly in charge of remotely monitoring and controlling various types of sensing equipment under the sea, according to real-time and dynamic observatory demands. Commands were reversely transmitted in the same communication channel and under the same communication protocols as how data was transmitted to ESOCS. The junction box played a role in assigning these commands to specific underwater sensors to control their working status (as shown by the red information flow in Figure 5). 

### 3.2. Plug-and-Play Development

In the context of ESOCS monitoring architecture, plug-and-play was respectively enabled in the networking layer and the service layer. On the one hand, the general junction box (GJB) designed in the networking layer could dynamically interface and network marine sensors attached. On the other hand, the ocean sensor markup language (OSML) implemented in the service layer could model sensor information in a standardized way and contribute to the remote hot swapping control of in situ sensors. The GJB-OSML enabled control method (GOE Control Method) was thus proposed as the plug-and-play solution for implementing ESOCS [22]. In this way, the remote client in the application layer could instantly control any newly added sensor in the sensing layer. 

**Figure 6:**
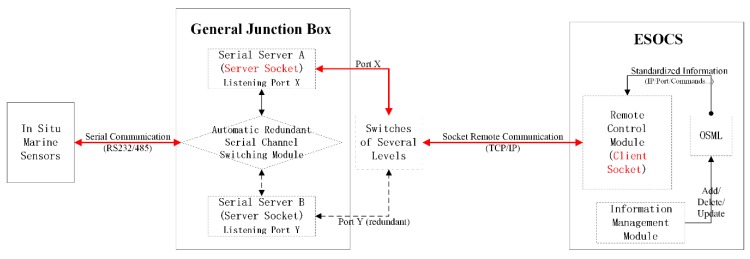
Plug and Play Enablement.

As shown in Figure 6, the GOE Control Method was detailed in the following way. On the one hand, the general junction box (GJB) was integrated with a smart serial server that used the TCP/IP protocol and worked in a server socket mode. The server was given a standalone IP address and every physical port corresponded to a unique port number. Thus, every ocean sensor attached to GJB was automatically allocated a unique pair of IP address/port number and was represented as a Sensing Endpoint in the network. Once a socket connection was established with a unique pair of IP/port information offered by a Sensing Endpoint, the sensor attached to that corresponding physical port was exposed on the Internet and the interactive transmission of observation data/control commands can be performed using this connection. GJB accordingly succeeded in interfacing ocean sensors in cabled seafloor observatories and networking them as corresponding Sensing Endpoints ready for bidirectional remote communications. 

On the other hand, OSML was designed with customized elements that both precisely defined functional requirements laid for ESOCS and described various types of information for controlling/managing in situ sensors. With OSML properly marked up and its elements properly nested, component-oriented modules of ESOCS can process the OSML tags in a standardized and dynamic way as follows (Figure 6). Once any current sensor needed reconfiguration or any new sensor was added, the Information Management Module of ESOCS called corresponding methods to update instantiated OSML files without affecting the current operations of the whole system. The Remote Control Module of ESOCS then loaded the updated information such as IP address/port number, established remote connections, and performed control operations on reconfigured or newly added sensors without interfering with other in situ sensing equipment. This design enabled a better remote control and management of cabled observatory sensor systems in the ECS.

With the proposed monitoring architecture and GOE Control Method, ESOCS was developed as a plug-and-play and module-integrated control system. Based on the configuration principle that modules should be mixed randomly, a smart serial server was introduced in the general junction box to act as the server component for listening, connecting, receiving and sending data. In this case, ESOCS was mainly implemented as a client program structured into five modules, namely, a remote control module, an information retrieval module, an information management module, a system management module and a user management module. The remote control module was responsible for the remote communication and dynamic control process, while the information management module took on the process of managing sensor control information modeled by OSML and updating the information in both foreground interfaces and background database (Figure 6). Other modules functioned and contributed to a robust and complete sensor control system for cabled seafloor observatories.

### 3.3. Case Study

#### 3.3.1. Experimental Scenario: The Xiaoqushan Seafloor Observatory

**Figure 7:**
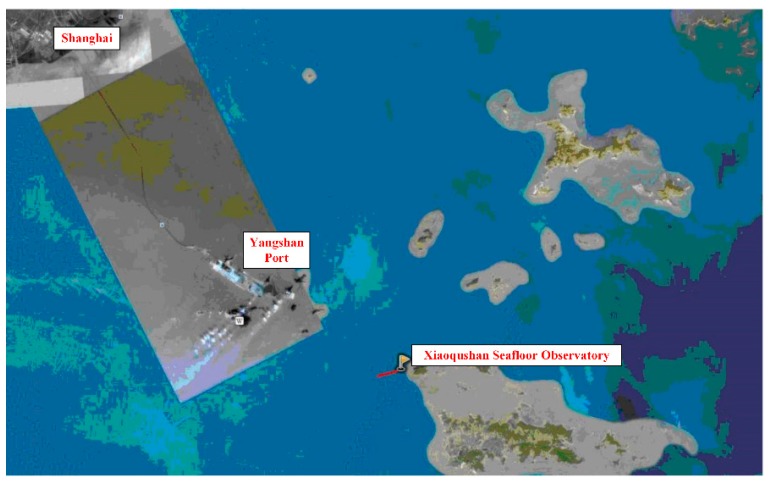
Location of the Xiaoqushan Seafloor Observatory.

The Xiaoqushan Seafloor Observatory is established in the inner shelf off Shanghai (Figure 7), geographically between 30°31′44″ N, 122°15′12″ E and 30°31′34″ N, 122°14′40″ E. The focused area marks the confluence of the Yangtze River and the Open Ocean and configures strong land-sea interactions. As a coastal cabled observatory, the Xiaoqushan Seafloor Observatory originally consisted of a double-armored composite optical cable that was 1.1 km long, a special junction box, an ADCP sensor, a CTD sensor, an OBS sensor and an anti-trawl frame. Observatory upgrades started in October 2011 and lasted for 21 months. The first and most important upgrade was the general junction box enabled to interface various types of marine sensors in a plug-and-play way, transforming the observatory into a real test bed for oceanographic instrumentation. The second upgrade was the integration of more multidisciplinary sensors into the observatory. The third upgrade was a series of reconstruction work to handle the increasing amount of transmitted data. Since it was first put into service in April 2009, the Xiaoqushan Seafloor Observatory has been performing continuous measurements and satisfactory operations for more than six years. This successful observatory lays a solid foundation for accumulating networking technologies under the sea, and contributes to the plan for more integrated and advanced cabled seafloor observatories in the ECS.

#### 3.3.2. Prototype System Test: Processes and Results

**Figure 8:**
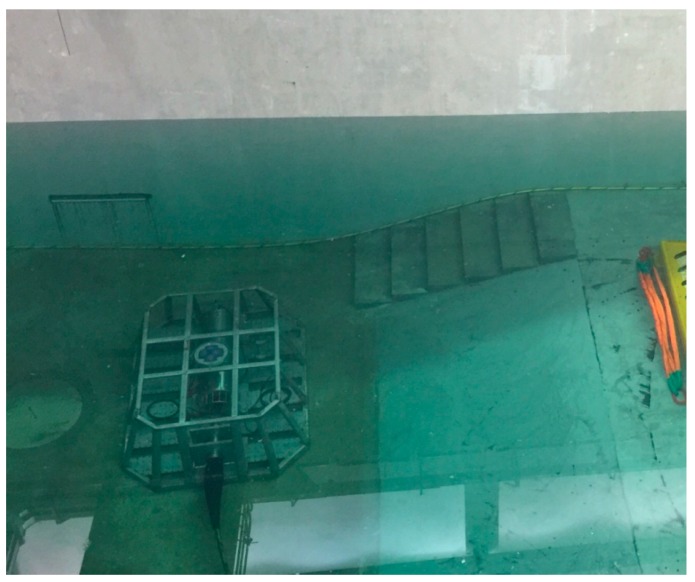
Tank Test for ESOCS.

**Figure 9:**
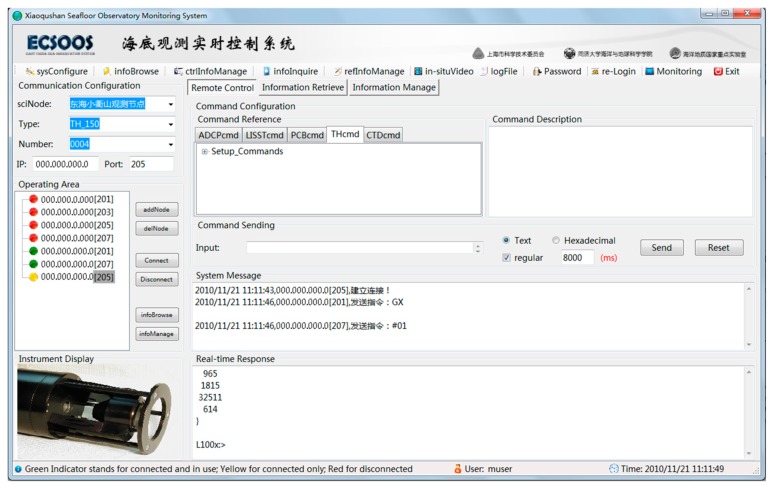
Main Interface of ESOCS.

As a prototype control system derived from the sensor control model, ESOCS was tested for its plug-and-play enablement through a series of trials. Individual module tests were first performed on the remote control module and other modules of ESOCS. At this stage, ESOCS was tested to ensure that all modules meet functional and performance requirements such as functional integrity, reliability (result-running accuracy and error-handling capabilities) and adaptability (processing capacity for changes in demand). All the modules were then integrated into ESOCS for a tank test in the seafloor observatory laboratory at Tongji University, in the process of which a general junction box and several types of marine sensors were connected and simulated as the in situ observatory (Figure 8). The whole system was tested for all the function points and was continuously debugged under both normal operations and boundary conditions. The tank test proved that ESOCS was able to synchronously control multiple sensors in a plug-and-play way and comply with all expected requirements, maintaining an uninterrupted operation for as long as 2000 h. ESOCS was finally tested during a shallow sea trial at the Xiaoqushan Seafloor Observatory before it was put into service and proved to function well during the entire sea trial program. The test of sensor control process was shown in the main interface of ESOCS (Figure 9).

Since it was put into use, the control model-derived ESOCS has been functioning consistently and presenting satisfactory practical performance in accordance with all the requirements laid for the operational Xiaoqushan Seafloor Observatory: (i) all the deployed marine sensors and observatory equipment were remotely controllable and on-line monitored; (ii) all the in situ observation data were received, interpreted and stored with associated metadata in a real-time way; (iii) the control model derived ESOCS architecture enabled interface with other observation systems such as autonomous underwater vehicles and different kinds of moorings or cable-shore systems; (iv) the Xiaoqushan Seafloor Observatory supported both commercial and customized oceanographic instrumentation for shallow water trial and the experimental process was simple and robust. 

Given the hostility of the marine environment in the ECS to in situ observatory equipment, the sensor control model derived ESOCS guaranteed that the Xiaoqushan Seafloor Observatory could provide datasets of over six-year duration to contribute a continuous and adaptive record for studying sea-land interactions in the focused area. The available record showed clearly that there was considerable variability on time scales that were not captured by a routine boat survey. An initial analysis of some in situ observation data sets from the Xiaoqushan Seafloor Observatory demonstrated that long-term measurements contributed to comprehensive studies on the mechanisms of ocean dynamics and environmental variations under different weather conditions [23]. Another initial outcome was the observatory data record and analysis of the tsunami induced by the 2010 Chilean earthquake [32], which also confirmed the practical performance of ESOCS from the perspective of data acquisition and application.

The case study showed that the proposed sensor control model was effective in designing, developing and deploying (3D) a sensor control system for cabled seafloor observatories. The monitoring architecture of ESOCS was designed and derived from the first deployment scenario of the sensor control model, and was mapped to all the functional layers of the model. Based on the architecture, ESOCS was developed as a plug-and-play and module-integrated control system. ESOCS was tested and deployed to the Xiaoqushan Seafloor Observatory, which was consistently functioning and presenting satisfactory practical performance in accordance with all the requirements laid for the operational observatory. The abstract sensor control model was thus proved its effectiveness by all the 3D instantiation processes as stated above in Section 3.

## 4. Discussion and Conclusions

In this paper, a sensor control model for cabled seafloor observatories is proposed. The model abstracts the sensor control as a standardized bidirectional information flow process and accordingly structures a sensor control system into the application layer, the service layer, the networking layer, and the sensing layer. Layered functions and interactions between adjacent layers in return realize this information flow process. A prototype control system is implemented, the monitoring architecture of which is derived from one of the deployment scenarios of the sensor control model. The prototype control system has been tested for its plug-and-play enablement through a series of trials, and has been consistently functioning and presenting satisfactory practical performance in accordance with all the requirements laid for the operational Xiaoqushan Seafloor Observatory.

The model divides the sensor control system into functionally independent layers, of which the adjacent upper layer passes parameters to the adjacent bottom layer and the adjacent bottom layer provides services to the adjacent upper layer. Layers interact via standard interfaces to fulfill all the information exchange processes throughout the lifecycle of the two information flows within the control system. From the view of control command flow, the application layer first generates control demands. Facing these demands, the service layer calls functional services to operate related information and produce control plans (namely formatted control commands). The networking layer then receives the control commands transmitted over the network and translates them into serially transmitted information for the sensing layer. The sensing layer finally executes these control commands and returns response data. From the view of observation data flow, the sensing layer first senses the marine environment and obtains in situ observation data. The networking layer then collects all the serially-transmitted data, converts them into network data stream and transmits them to the service layer. The service layer calls related services to receive and store observation data, and to return response information to the application layer. The application layer finally produces new sensor control requirements in terms of related data analysis and application results.

Based on this division, the sensor control model provides a theoretical specification for designing, developing and deploying (3D) a sensor control system for cabled seafloor observatories. In practical applications, 3D modelling process can be explained in detail as follows. Firstly, the deployment scenario of the control model can be confirmed in accordance with specific engineering requirements and resource configurations. Secondly, the monitoring architecture can be designed and derived from the deployment scenario in terms of hardware/software elements in each layer. Thirdly, all the modules can be developed and integrated into the whole prototype control system for a series of trials. Finally, the control system can be deployed and applied to operational cabled seafloor observatories for testing and accomplishing all the functional requirements laid for the project. In this way the sensor control model contributes to the vertical management and observatory control of an operational cabled seafloor observatory, and enables that all the control system elements perform their own functions and interact with each other in a standardized way. In addition, the proposed sensor control model could be extended to offshore platforms such as mentioned in [33,34,35].

To summarize, major contributions of the paper are: (i) A sensor control model for cabled seafloor observatories. The model is information oriented and divides sensor control systems into functionally independent layers interacted in a standardized way. The model provides a standard theory for designing, developing and deploying a sensor control system for cabled seafloor observatories. The model contributes to both vertical management and horizontal integration of operational seafloor observatory sensor networks, and could be extended to offshore platforms. (ii) A prototype control system, ESOCS. The monitoring architecture of ESOCS is derived from the sensor control model and mapped to all the functional layers. The architecture contributes to a system level of remotely controlling observatory sensors in a standardized way, based on which ESOCS is designed and developed with plug-and-play enablement. (iii) A case study of the ESOCS deployment and application. The case study contains a series of trials for ESOCS and the application scenario is made for the Xiaoqushan Seafloor Observatory. All the successful operational processes of ESOCS and initial outcomes mark the effectiveness of the sensor control model. Future work shall include sensor control model optimization and characteristic analysis, and studies on other deployment scenarios of the model.
